# Histamine, mast cell tryptase and post-exercise hypotension in healthy and collapsed marathon runners

**DOI:** 10.1007/s00421-021-04645-0

**Published:** 2021-02-24

**Authors:** I. T. Parsons, M. J. Stacey, L. Faconti, N. Hill, J. O’Hara, E. Walter, B. Farukh, R. McNally, H. Sharp, A. Patten, R. Grimaldi, N. Gall, P. Chowienczyk, D. R. Woods

**Affiliations:** 1 Research and Clinical Innovation, Royal Centre for Defence Medicine, Birmingham, England; 2 School of Cardiovascular Medicine and Sciences, King’s College London, London, UK; 3 Imperial College Healthcare NHS Trust, London, UK; 4 Carnegie School of Sport, Leeds Beckett University, Leeds, England; 5 King’s College Hospital, London, UK; 6 Royal Surrey County Hospital NHS Foundation Trust, London, UK; 7 Brighton and Sussex NHS Trust, London, UK

**Keywords:** Anaphylaxis, Mast cells, Basophils, Exercise-associated collapse, Exertional heat illness, Heat stroke, Tryptase, Syncope, Degranulation

## Abstract

**Purpose:**

Heat stress exacerbates post-exercise hypotension (PEH) and cardiovascular disturbances from elevated body temperature may contribute to exertion-related incapacity. Mast cell degranulation and muscle mass are possible modifiers, though these hypotheses lack practical evidence. This study had three aims: (1) to characterise pre–post-responses in histamine and mast cell tryptase (MCT), (2) to investigate relationships between whole body muscle mass (WBMM) and changes in blood pressure post-marathon, (3) to identify any differences in incapacitated runners.

**Methods:**

24 recreational runners were recruited and successfully completed the 2019 Brighton Marathon (COMPLETION). WBMM was measured at baseline. A further eight participants were recruited from incapacitated runners (COLLAPSE). Histamine, MCT, blood pressure, heart rate, body temperature and echocardiographic measures were taken before and after exercise (COMPLETION) and upon incapacitation (COLLAPSE).

**Results:**

In completion, MCT increased by nearly 50% from baseline (*p* = 0.0049), whereas histamine and body temperature did not vary (*p* > 0.946). Systolic (SBP), diastolic (DBP) and mean (MAP) arterial blood pressures and systemic vascular resistance (SVR) declined (*p* < 0.019). WBMM negatively correlated with $\Delta$ SBP (*r* = − 0.43, *p* = 0.046). For collapse versus completion, there were significant elevations in MCT (1.77 ± 0.25 μg/L vs 1.18 ± 0.43 μg/L, *p* = 0.001) and body temperature (39.8 ± 1.3 °C vs 36.2 ± 0.8 °C, *p* < 0.0001) with a non-significant rise in histamine (9.6 ± 17.9 μg/L vs 13.7 ± 33.9 μg/L, *p* = 0.107) and significantly lower MAP, DBP and SVR (*p* < 0.033).

**Conclusion:**

These data support the hypothesis that mast cell degranulation is a vasodilatory mechanism underlying PEH and exercise associated collapse. The magnitude of PEH is inversely proportional to the muscle mass and enhanced by concomitant body heating.

**Supplementary Information:**

The online version contains supplementary material available at 10.1007/s00421-021-04645-0.

## Introduction

Light headedness, faintness, dizziness or collapse are common causes for presentation to medical facilities during, and after, marathon races (Roberts 2000, 2007). The aetiology of incapacitation in these circumstances is thought to include exaggerated post-exercise hypotension due to pooling of blood in the lower extremities, secondary to decreased vascular resistance in conjunction with resetting of the baroreflex (Asplund et al. 2011; Halliwill et al. 2015). Post-exercise hypotension is characterised by a persistent drop in systemic vascular resistance (SVR), in the absence of adequate increases in cardiac output (CO) to match pre-exercise blood pressure. Loss of the “muscle pump” upon cessation of exercise, coupled with exertional hypohydration, and increased venous pooling leads to a reduction in central venous pressure (CVP) and cardiac filling(Halliwill et al. 2000). In healthy individuals, during exercise, blood pressure increases proportional to workload (Iellamo 2001) despite a sustained vasodilation observed within skeletal muscle(Harold Laughlin et al. 2012; Halliwill et al. 2013, 2015) demonstrated following whole body exercise and after exercising a smaller muscle mass(Halliwill et al. 1996; Barrett-O’Keefe et al. 2013). However, it is unknown if the whole body muscle mass (WBMM) (absolute and as a percentage of body mass) correlates with the degree of vasodilation, and subsequent post-exercise hypotension.

Combined H_1_ and H_2_ receptor antagonism has been shown to significantly reduce post-exertional vasodilation(Lockwood et al. 2005; McCord et al. 2006; McCord and Halliwill 2006) which has led to the hypothesis that histamine may be a significant compound driving vasodilation and, consequently, post-exertional hypotension(Halliwill et al. 2013). Histamine is a biogenic amine, with potent vasoactive effects. It is generated intracellularly from histadine by histadine decarboxylase, with primary storage in the secretory granules of mast cells and basophils. Tryptase, commonly known as mast cell tryptase (MCT), is relatively specific for histamine release and coincident with mast cell activation (Laroche et al. 1992). Elevated plasma or serum MCT levels, therefore, indicate mast cell activation, degranulation and histamine release into the extracellular environment (Kabashima et al. 2018). MCT is commonly used to support the clinical diagnosis of anaphylaxis where MCT rise, due to widespread mast cell degranulation, correlates with the magnitude of hypotension and may persist in the blood for several hours (Dua et al. 2018). In the context of exercise, plasma MCT has been studied in the context of exercise-induced anaphylaxis (Schwartz 1995), a rare condition where anaphylaxis is associated with physical exertion.

Post-exercise hypotension is exacerbated by heat stress (Rivas et al. 2019) from strenuous or prolonged muscular exertion, and may result in relatively reduced skin blood flow and sweating (Kenny and McGinn 2017), so delay the expected restoration of thermoregulation following cessation of exertional metabolic heat production. Histamine is also released during exercise (Lockwood et al. 2005) and heat may be causal in mast cell degranulation (Luttrell and Halliwill 2017). Histaminergic vasodilation may, therefore, be contributory to instances of collapse during exercise such as exertional heat illness.

We hypothesised that marathon running would associate with elevated MCT, and possibly histamine, detectable in the systemic circulation and that this would be reflected in cardiovascular responses observed with exercise. We were also interested to explore whether such changes would vary in collapsed runners, who may be subject to similar but potentially more pronounced physiological responses in the context of hyperthermia contributing to incapacity. We further hypothesised that muscle mass (absolute and as a percentage of body mass) would predict changes in histamine, MCT or blood pressure.

Therefore, this study had three distinct aims: (1) to demonstrate differences in mast cell tryptase (MCT) and/or histamine in healthy runners following completion of a marathon, in comparison to resting samples. (2) To investigate relationships between muscle mass and changes in blood pressure. (3) To identify differences in histamine and MCT in collapsed runners in comparison to healthy runners who completed the race.

## Methods

### Experimental design

A prospective cohort study of recreational runners were recruited prior to the 2019 Brighton Marathon (COMPLETION group) following ethical approval (London South East ethics committee (19/LO/0340 247967). Blood samples, echocardiography, physiological measurements and body composition analysis was performed. All measures were performed in the 48 h preceding the start of the marathon (COMPLETION-pre) and repeated immediately following the race completion (COMPLETION-post), with the exception of body composition (rested baseline only). Blood analysis was repeated 4 h post-marathon and 24 h post-marathon where possible. A further case–control study was performed where collapsed runners were recruited (COLLAPSE group) who had equivalent measures taken as soon as possible (< 30 min) post-collapse. The collapse group was compared to the COMPLETION-post. The local ambient temperature on the day was 8 °C increasing to 12 °C over the timeframe of the marathon event with a humidity range of 49–78%.

### Participants

Overall 24 recreational runners were recruited (COMPLETION group). No participants were taking antihistamines. 23 participants self-identified as being ‘White British’ with 1 participant self-identifying as ‘Mixed Race-Asian’. Six participants were additionally available for blood sampling at 4 h post-run and four participants had measures at 24 h following marathon completion. Participants with a history of ‘heat illness, exercise‐induced urticaria, exercise‐induced anaphylaxis or exercise‐induced hypersensitivity syndromes’ were excluded. Eight participants were recruited from among runners treated for collapse on the course (COLLAPSE group), or close to the finish line.

### Blood samples

Blood samples was drawn at the antecubital fossa. Whole blood was immediately centrifuged, and the plasma extracted and immediately frozen in liquid nitrogen and stored at – 86 °C until analysis (Affinity Biomarker Labs, London, UK). Histamine were measured using solid-phase enzyme-linked immunosorbent assay (ELISA) kits (Tecan, Reading, UK). MCT levels were measured using a sandwich ELISA (Abbexa Ltd, Cambridge, UK). The intra-assay precision range of the histamine assay was 0.5–85 μg/L [coefficient of variability (CV) 2.2–9.2%] and an inter-assay precision range of 7.6–86 μg/L (CV 6–13.8%) The intra-assay and inter-assay precision range of the MCT assay was 1.5–12 μg/L and a CV of < 10% and < 12%, respectively.

### Transthoracic echocardiography

Prior to marathon, and immediately on finishing, COMPLETION participants underwent a resting transthoracic echocardiogram (TTE) (CX50, Phillips, Amsterdam, Netherlands) performed by two blinded British Society of Echocardiography or European Society of Cardiology accredited practitioners. Post-processing was performed using Xcelera (Phillips, Amsterdam, Netherlands). TTE was performed in the partial left decubitus position except for subcostal views. A parasternal long-axis image was recorded with measurement the LVOT diameter. Left ventricular stroke volume (SV) was calculated in at least one of two ways: (1) from the product of the velocity–time integral (cm) of the pulsed-wave Doppler in the left ventricular outflow tract (LVOT) and the LVOT cross-sectional area (πr^2^ in cm^2^), determined by a TTE measurement of the LVOT in the parasternal long-axis view; (2) From the subtraction of the left ventricular end systolic volume (LVESV) from the left ventricular end diastolic volume (LVEDV), calculated using Simpson biplane method (Wharton et al. 2015), in both two-chamber and four-chamber views, where possible, and then averaged. The maximal inferior vena cava diameter was recorded and the degree of respiratory collapse was noted with the collapsibility index calculated. The left atrial volume was measured in four-chamber view, and where possible, two-chamber view. The right atrial volume were measured in four-chamber view. We estimated the right atrial pressure by the inferior vena cava (IVC) diameter and collapsibility only; as outlined by the American Society of Echocardiography (Lang et al. 2015). Where the IVC was ≤ 2.1 and collapsed > 50% a right atrial pressure (RAP) of 3 mmHg was given. Where the IVC was > 2.1 cm and collapsed < 50% a RAP of 15 mmHg was given. Where the IVC diameter and collapsibility did not fit this criteria a RAP of 8 mmHg was given. For the COLLAPSE group, measures were performed as soon as possible following incapacitation. No baseline measures were performed in the COLLAPSE group.

### Physiological measurements and body composition analysis

Heart rate (HR), systolic (SBP) blood pressure, diastolic blood pressure (DBP) peripheral oxygen saturations (SpO_2_) were measured with the participants lying at rest for 5 min (GE Carescape V100, UK). MAP was calculated from the SBP and DBP and corrected for HR [DBP + 0.33 + (HR × 0.0012) x (SBP-DBP)] (Razminia et al. 2004). Tympanic temperature was measured (Braun Thermoscan 3020, Kronberg, Germany) and height was recorded barefooted using a stadiometer. In the COLLAPSE group, a core (rectal) temperature measurement was taken. CO was calculated by multiplying the SV (VTI x LVOT cross-sectional area) by the HR. The cardiac index (CI) was calculated by dividing the CO by the body surface area (BSA) (Mosteller). The SVR was calculated by 80x (MAP-CVP)/CO with the SVR index (SVRI) calculated by 80x (MAP-CVP/CI). For the purposes of the study, the TTE estimate RAP was considered analogous to central venous pressure. Body composition was measured by bioelectrical impedance (Tanita, MC-780MA P) also barefooted with minimal clothing with WBMM recorded as absolutes and as a proportion (%).

### Statistical analysis

Mast cell tryptase has not been measured in relation to marathon running, but the mean and standard deviation of tryptase in normal participants in a study of 56 participants (Schwartz et al. 1994) was 4.9 ± 2.3 μg/L. We, therefore, calculated that 21 participants would detect a 40% rise in tryptase (alpha 0.05, beta 0.8). As recruitment to the study was dependent upon the dynamic availability of collapse cases, a formal power calculation for comparison with completion was not undertaken.

Measures were assessed for normality using the Shapiro–Wilk test prior to data analysis. For the primary aim, pre- and post-measures in the completion group were compared using a repeated measures one-way ANOVA of selected pairs (mixed effects model to account for any partially complete data) with correction for multiple comparisons (Holm-Šídák). For 4 h and 24 h, MCT measures a paired Student’s *t* test was performed comparing the delayed measures (4 h and 24 h) with baseline measures. For Aim 2, post-marathon collapse measures were compared to corresponding completion measures using an unpaired Student’s *t* test for parametric values and Mann–Whitney *U* test for non-parametric values. For Aim 3, correlation analysis was performed for muscle mass (absolute and proportion of total body mass) with SBP, DBP and MAP. Values were expressed as mean and standard deviation. The *α* level was set to 0.05. All statistical analyses were performed using GraphPad Prism 8.0, GraphPad Software, San Diego, California.

## Results

COMPLETION participants were 39 ± 9 years old, of whom 10 (42%) were female and 14 (58%) male. This group finished the marathon in 252 (4.2 h) ± 42 min. Body mass index pre-marathon was 24.0 ± 2.5 kg/m^2^. Pre- and post-marathon comparison can be seen in Table 1. In the COMPLETION group, six participants were available to attend for further blood samples, MCT remained significantly elevated at 4 h (0.87 ± 0.32 μg/L) versus rested baseline (0.58 ± 0.33 μg) *p* = 0.040. In four participants, the MCT remained elevated at 24 h (0.88 ± 042 μg/L) compared to baseline but this was not significant (*p* = 0.668).

**Table 1:** A comparison of pre- and post-marathon histamine, mast cell tryptase, physiological observations, and echocardiographic derived measures

		Baseline	Post marathon	*p* value
		Mean (SD)	Mean (SD)	
Blood plasma				
Histamine	(μg/L)	0.46 (0.37)	13.7(33.9)	0.3887
Mast Cell Tryptase	(μg/L)	0.81 (0.39)	1.12 (0.43)	0.0049**
Physiological observations				
Resting heart rate	(/min)	57 (13)	85 (15)	< 0.0001****
Temperature	(°C)	36.2 (0.7)	36.2 (0.8)	0.9462
Systolic blood pressure	(mmHg)	126 (17)	113 (17)	0.0192*
Diastolic blood pressure	(mmHg)	81 (8)	72 (7)	0.0006***
Mean arterial pressure	(mmHg)	84 (8)	77 (8)	0.0024**
Echocardiographic-derived measures				
LVEDV/BSA	(ml/m^2^)	71 (12)	60 (14)	0.0234*
LVESV/BSA	(ml/m^2^)	29 (7)	24 (8)	0.0192*
LVSV/BSA (LVEDV-LVESV)	(ml/m^2^)	42 (6)	36 (8)	0.0008***
LVSV/BSA (LVOT area x LVOT VTI)	(ml/m^2^)	44 (9)	41 (9)	0.6725
Cardiac index	(L/m^2^)	2.44 (0.68)	3.52 (0.85)	0.0008***
Left atrial volume/BSA	(ml/m^2^)	24 (7)	21 (8)	0.3546
Right atrial volume/BSA	(ml/m^2^)	25 (10)	20 (9)	0.3887
IVC diameter (expiration)	(cm)	1.95 (0.49)	1.35 (0.45)	0.0003***
Central venous pressure	(cm)	8.6 (3.8)	6.4 (3.1)	0.2076
Systemic vascular resistance index	(dynes · sec/cm^5^/m^2^)	2669 (857)	1670 (451)	0.0008***

*LVEDV* left ventricular end diastolic volume, *LVESV* left ventricular end systolic volume, *BSA* body surface area (Mosteller), *LVOT* left ventricular outflow tract, *VTI* velocity time integer, *IVC* inferior vena cava

* ≤ 0.05

** ≤ 0.01

*** ≤ 0.001

**** ≤ 0.0001

**Fig. 1:**
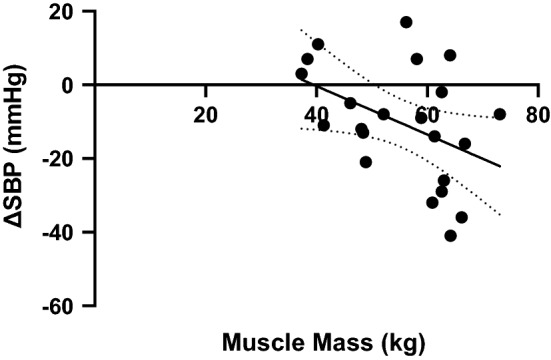
Linear regression of whole body muscle mass and Δ systolic blood pressure (*r*^2^ = 0.19) Equation: Δ systolic blood pressure = − 0.6582* total body muscle mass + 26.01). *SBP* systolic blood pressure (mmHg). Muscle mass (kg) as recorded by bioelectrical impedance

The mean body mass of the COMPLETION group was 73.5 ± 10.5 kg pre-marathon and 70.7 ± 9.8 post-marathon (*p* < 0.0001) with a mean Δ of − 1.72 ± 1.2 kg (2.3%). The mean WBMM was 55.4 ± 10.1 kg with a mean %WBMM of 75.1 ± 7%. On correlating absolute WBMM, there was a significant negative correlation with ΔSBP (*r* = − 0.43, *p* = 0.046) (Fig. 1). The %WBMM was also negatively correlated with ΔSBP (*r* = − 0.23) but this was not significant (*p* = 0.30). There was also non-significant negative correlation with WBMM and ΔDBP (WBMM: *r* = − 0.37, *p* = 0.090, %WBMM: *r* = − 0.36 *p* = 0.097) or ΔMAP (WBMM: *r* = − 0.38 *p* = 0.085, %WBMM *r* = − 0.33 *p* = 0.133).

When comparing the change in BP (COMPLETION-post—COMPLETION-pre) by sex, there was a significant differences in mean DBP (men − 12.6 mmHg; women − 4.44 mmHg *p* = 0.045), MAP (men − 11.1 mmHg, women − 3.11 mmHg, *p* = 0.047) but not SBP (men − 13.7 mmHg, women − 5.78 mmHg, *p* = 0.52) (two-way ANOVA mixed effects model with Šídák's correction for multiple comparisons). There was no significant difference between men and women in terms of the magnitude of MCT or histamine rise post-marathon.

**Fig. 2:**
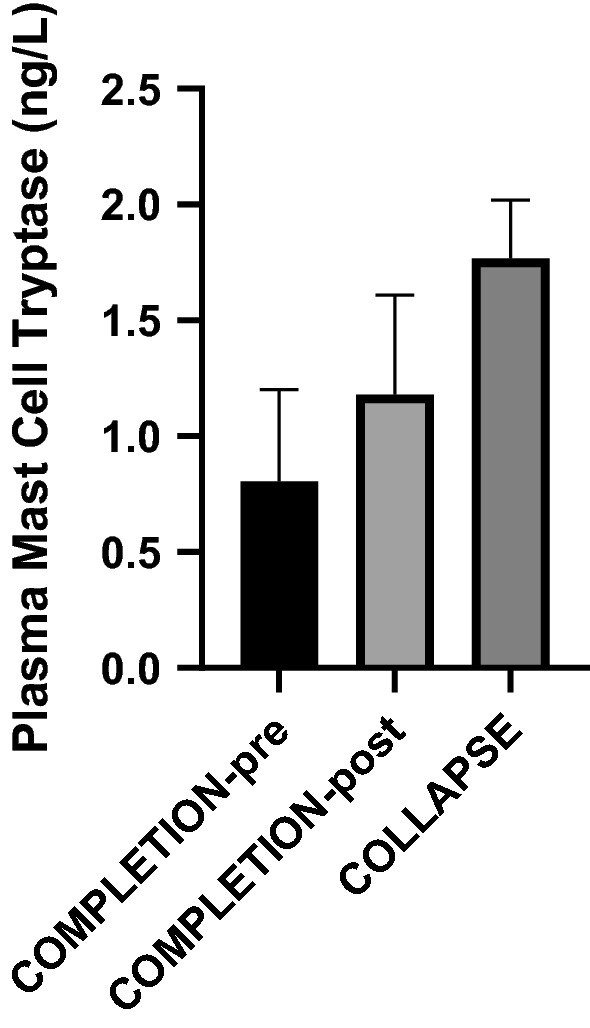
Plasma mast cell tryptase levels in COMPLETION-pre (*n* = 24, *p* = 0.0049) and COLLAPSE (*n* = 8, *p* = 0.001) compared to COMPLETION-post

Of the eight COLLAPSE participants, five were male (62.5%), three were female (37.5%). The COLLAPSE group’s post-marathon results are compared to COMPLETION in Table 2. Overall 5/8 participants (62.5%) underwent echocardiography. MCT measured in COLLAPSE exceeded both pre- and post-marathon values in COMPLETION (Fig. 2).

**Table 2:** A comparison of participants of post-marathon values of participants who completed the marathon (COMPLETION-post) with participants who collapsed during, or following, the marathon completion (COLLAPSE)

		Collapse	Completion-post	*P* value
		Mean (SD)	Mean (SD)	
Blood plasma				
Histamine	(μg/L)	9.6 (17.9)	13.7 (33.9)	0.1074
Mast cell tryptase	(μg/L)	1.77 (0.25)	1.18 (0.43)	0.0010**
Physiological observations				
Resting heart rate	(/min)	124 (23)	85 (15)	< 0.0001****
Temperature	(°C)	39.8 (1.3)	36.2 (0.8)	< 0.0001****
Systolic blood pressure	(mmHg)	115 (13)	113 (17)	0.8452
Diastolic blood pressure	(mmHg)	60 (9)	72 (7)	0.0012**
Mean arterial pressure	(mmHg)	68 (9)	77 (8)	0.0327*
Echocardiographic-derived measures				
Systemic vascular resistance	(dynes · sec/cm^5^)	537 (197)	898 (245)	0.0049**
Stroke volume	ml	84 (25)	78 (17)	0.4986
Cardiac output	L/m	10.0 (3.5)	6.5 (1.7)	0.002**

* ≤ 0.05

** ≤ 0.01

*** ≤ 0.001

**** ≤ 0.0001

## Discussion

This is the first study to establish that marathon running increases plasma MCT. In addition, MCT was shown to be more pronounced in runners collapsing with relative hypotension and hyperthermia versus successful finishers. To our knowledge, it is also the first to demonstrate a relationship between WBMM and the magnitude of post-exertional hypotension. The novel increase in MCT, in the healthy runners who completed the marathon, is likely derived from degranulation of mast cells associated with the exercising muscle and its vascular supply (Metcalfe et al. 1997). In this sense, the measures recorded upon completion of the marathon may have represented ‘spill-over’ from local tissue mast cell populations, with the relatively greater half-life and reduced clearance of MCT versus histamine facilitating observation of the former, but not the latter, in blood drawn from the venous circulation.

These data support the hypothesis that mast cell degranulation contributes to cardiovascular changes with exercise. A previous study has shown elevations post-exercise in interstitial tryptase and histamine in skeletal muscle (Romero et al. 2017). We were unable to find a significant increase in histamine post-marathon in comparison to baseline or on comparing COMPLETION-post group to COLLAPSE. This is in keeping with other studies which have found the rise in plasma histamine difficult to demonstrate (Halliwill et al. 1996; Ely et al. 2017), although less contemporary studies have shown a histamine rise (Dunér and Pernow 1958; Harries et al. 1979). Difficulties in measuring histamine are thought to be due to the inherent short half-life of plasma histamine, with rapid diffusion (Schwartz et al. 1989; Laroche et al. 1992), which predominantly exerts a local effect in skeletal muscle tissue (Romero et al. 2017). While we attempted to take blood as soon as possible following race completion in the COMPLETION group and as soon as possible following collapse in the COLLAPSE group, there was an unavoidable non-standard delay which could have influenced the accuracy of histamine levels. The MCT concentration can be considered more robust and still significantly elevated at 4 h post-race completion.

The correlation between the change in SBP and baseline muscle mass supports muscle-associated mechanisms in mediating the haemodynamic response post-marathon, for which there are numerous other explicatory candidates. Factors associated with immediate post-exercise hyperaemia are considered less dependent upon the condition set by the work done by the muscles which, in whole body exercise, may be reflected in muscle mass (Morganroth et al. 1975; Bangsbo and Hellsten 1998). However, mechanisms such as baroreflex resetting have been postulated to be directly proportional to the muscle mass involved in exercise (Halliwill et al. 2013). This would be consistent with our post-marathon sampling window capturing the sustained period of post-exercise vasodilatation known to follow moderate intensity and last in excess of 2 h, rather than the acute hyperaemic phase of post-exercise hypotension. Whilst there was no significant correlation with MAP and DBP the trend was, as with SBP, negatively correlated with WBMM. We did find a statistically significant increased difference (COMPLETION-post—COMPLETION-pre) in ΔDBP and ΔMAP in men, compared to women. In a novel model of post-exercise syncope Lacewell et al. found a significantly increased risk of syncope in men compared to women with the cause of tilt-test termination being hypotension in conjunction with pre-syncopal symptoms (Lacewell et al. 2014). Given the known sex differences in body composition this would support the hypothesis that a greater muscle mass results in greater post-exertional vasodilation, although this was not supported by differing MCT levels in men compared to women.

We detected a significant decrease in IVC diameter with marathon participation in COMPLETION, which along with the significant mean change of − 1.7 kg in body mass would suggest a degree of hypohydration post-marathon, although this was within prescribed limits for safe endurance exercise performance (Sawka et al. 2007). In a study of endurance, runners competing in an 80 km footrace Holtzhausen and Noakes found the degree of postural variation in blood pressure was unrelated to the degree of hypohydration (mean 4.6%) (Holtzhausen and Noakes 1995). Overall, this would support the hypothesis that the decrease in blood pressure values from pre to post, as seen in the COMPLETION group, are predominantly driven by exercise-induced vasodilatory and baroreceptor changes with hyperthermia or hypohydration being secondary factors (Asplund et al. 2011). This is supported by the echocardiographic data where there was significant decrease in SVRI (calculated from the reduced MAP, CVP and CI) in COMPLETION-post in comparison to COMPLETION-pre. Other factors that may play a role (Noakes 2007) include impaired sympathetic vascular regulation (Halliwill et al. 1996) and impaired cerebral autoregulation (Williamson et al. 2004; Carter et al. 2006).

In keeping with the significantly elevated core temperature in COLLAPSE vs COMPLETION-post, the working clinical diagnosis assigned to COLLAPSE cases was exertional heat illness. In health, the return of heat production to baseline rate with cessation of exercise is paradoxically met with abrupt centrally mediated suppression of heat loss from resetting of thermoregulatory reflexes, resulting in a sustained elevation in muscle and core temperature (Kenny and McGinn 2017). The post-exertional pooling of blood in the muscles of the lower limbs post-exercise reduces heat exchange resulting in the storing of heat in previously active muscles (Brotherhood 2008). This can maintain an elevated core temperature via convective exchange between blood and muscle (Brotherhood 2008) despite removal of the exertional metabolic heat stimulus. High metabolic and/or environmental heat loads exacerbate muscular heat content, and subsequent core temperatures, which can translate into significant post-exercise hypotension (Keyzer et al. 1984; Kenny and McGinn 2017) and thermal strain (Brotherhood 2008). The alteration in post-exercise thermoregulatory function has been associated with resetting of the baroreflex, which also contributes to systemic vasodilation and a pooling of blood in the extremities resulting in reductions in blood pressure (Halliwill et al. 2013). Mast cell degranulation contributes to cardiovascular changes with exercise and the present study indicates that these mechanisms may predispose or complicate supervening exertional heat illness as part of a continuum spanning post-exertional hypotension to exertional heat illness. Elevated temperature has also been implicated as a mechanism of mast cell degranulation (Halliwill et al. 2015; Luttrell and Halliwill 2017).

Urinary histamine and its metabolites have also been reported in the presence of histamine-producing bacteria in the gastrointestinal tract (Keyzer et al. 1984). Exercise is known to adversely disrupt the gastrointestinal barrier integrity (Parsons et al. 2019) and these bacteria may hypothetically play a role in the pathophysiology of heat stroke. In a study combining exercise (45 min at 50% VO_2_max) followed by 60° head-up tilt with randomised crossover between histamine blockade and control demonstrated that while blockade did not abolish the occurrence of post-exercise pre-syncope, there was a reduced incidence of hypotension and a trend towards lengthened time (94 s) to the onset of pre-syncope (McCord et al. 2008). In comparing COLLAPSE and COMPLETION-post groups, we also did not identify a difference in SV by echocardiography. We did identify a significant increase in CO in the COLLAPSE group in comparison to COMPLETION-post, driven by increased HR, and a significant reduction in SVR likely exaggerated by histaminergic vasodilation.

There are several limitations with regard to this study. We acknowledge that our COLLAPSE group was small and perhaps potentially underpowered, probably due to uncharacteristically cold weather (Holtzhausen and Noakes 1997), so will require further research to corroborate these data. While our measures were performed as close as possible to the point of collapse, or marathon completion, not all measurements were performed concurrently which will introduce confounding to the dynamic nature of post-exercise physiology. We hope to have mitigated the effect of this by taking samples within 30 min. There are no baseline data for the runners who subsequently collapsed as it was not possible to identify this group prospectively. Equally, we were unable to adequately characterise the COLLAPSE group in terms of their body composition and co-morbid state which may have confounded the findings, although focussed echocardiography ruled out structural heart disease as a cause in five participants. The working diagnosis in the COLLAPSE group was one of exertional heat illness as evidenced by the elevated core (rectal temperature). We did not seek to corroborate this using a diagnostic criteria or seek evidence of end organ damage other than the cardiovascular changes as shown in Table 2. For comparative purposes, we were only able to obtain tympanic temperatures in the COMPLETION group (Casa et al. 2007).

Echocardiographic measures have significant inter-observer and intra-observer variability (Hare et al. 2008) and echocardiographic derived measures are based on several assumptions. Whilst we made every effort to limit variability in the COMPLETION group echocardiographic measures the COLLAPSE group’s focussed imaging are likely to have been further compromised by missing measurements, poor acoustic windows and technical factors such as angular acuity of aortic blood flow, and/or off-axis aortic annular dimensions. TTE measures of SV, and subsequently CO and SVR are commonly underestimated due to assumptions in accurately measuring the LVOT (Chin et al. 2014). Whilst body composition using bioelectrical impedance is comparable to dual-energy X-ray absorptiometry, there is individual variability associated with bioelectrical impedance estimations for both single assessments and repeated measurements (Moon 2013).

## Conclusion

These data support the hypothesis that mast cell degranulation, represented by MCT, is a predominant vasodilatory mechanism driving post-exertional hypotension and also contributory to, or as a consequence of, exertional heat illness. These findings provide a new perspective on approaches to monitoring and interpreting mechanisms that favour post-exercise hypotension and exertional collapse, which may have practical applications in reducing medical presentations or even hospitalisation risk with marathon participation.

## Supplementary Information

Below is the link to the electronic supplementary material.

Supplementary file1 (DOCX 174 KB)

## Data Availability

All data available on request.

## Abbreviations

BSA: Body surface area
CI: Cardiac index
CO: Cardiac output
CVP: Central venous pressure
DBP: Diastolic blood pressure
ELISA: Enzyme-linked immunosorbent assay
HR: Heart rate
IVC: Inferior vena cava
LVEDV: Left ventricular end diastolic volume
LVESV: Left ventricular end systolic volume
LVOT: Left ventricular outflow tract
MAP: Mean arterial pressure
MCT: Mast cell tryptase
RAP: Right atrial pressure
SBP: Systolic blood pressure
SV: Stroke volume
SVR: Systemic vascular resistance
SVRI: Systemic vascular resistance index
TTE: Transthoracic echocardiography
VTI: Velocity–time integer
WBMM: Whole-body muscle mass
