# Correction to: Histamine, mast cell tryptase and post-exercise hypotension in healthy and collapsed marathon runners

**DOI:** 10.1007/s00421-021-04782-6

**Published:** 2021-08-19

**Authors:** I. T. Parsons, M. J. Stacey, L. Faconti, N. Hill, J. O’Hara, E. Walter, B. Farukh, R. McNally, H. Sharp, A. Patten, R. Grimaldi, N. Gall, P. Chowienczyk, D. R. Woods

**Affiliations:** 1 Research and Clinical Innovation, Royal Centre for Defence Medicine, Birmingham, England; 2 School of Cardiovascular Medicine and Sciences, King’s College London, London, UK; 3 Imperial College Healthcare NHS Trust, London, UK; 4 Carnegie School of Sport, Leeds Beckett University, Leeds, England; 5 King’s College Hospital, London, UK; 6 Royal Surrey County Hospital NHS Foundation Trust, London, UK; 7 Brighton and Sussex NHS Trust, London, UK

## Correction to: European Journal of Applied Physiology (2021) 121:1451–1459 10.1007/s00421-021-04645-0

The article Histamine, mast cell tryptase and post-exercise hypotension in healthy and collapsed marathon runners, written by I. T. Parsons, M. J. Stacey, L. Faconti, N. Hill, J. O’Hara, E. Walter, B. Farukh, R. McNally, H. Sharp, A. Patten, R. Grimaldi, N. Gall, P. Chowienczyk and D. R. Woods, was originally published Online First without Open Access. After publication in volume 121, issue 5, pages 1451–1459 the author decided to opt for Open Choice and to make the article an Open Access publication. Therefore, the copyright of the article has been changed to © The Author(s) 2021 and this article is licensed under a Creative Commons Attribution 4.0 International License, which permits use, sharing, adaptation, distribution and reproduction in any medium or format, as long as you give appropriate credit to the original author(s) and the source, provide a link to the Creative Commons licence, and indicate if changes were made. The images or other third party material in this article are included in the article’s Creative Commons licence, unless indicated otherwise in a credit line to the material. If material is not included in the article’s Creative Commons licence and your intended use is not permitted by statutory regulation or exceeds the permitted use, you will need to obtain permission directly from the copyright holder. To view a copy of this licence, visit http://creativecommons.org/licenses/by/4.0/.

The original article has been corrected.

